# 3-Bromophenyl 6-acetoxymethyl-2-oxo-2*H*-1-benzopyran-3-carboxylate inhibits cancer cell invasion *in vitro* and tumour growth *in vivo*

**DOI:** 10.1038/sj.bjc.6600856

**Published:** 2003-04-01

**Authors:** I Kempen, D Papapostolou, N Thierry, L Pochet, S Counerotte, B Masereel, J-M Foidart, M Reboud-Ravaux, A Noël, B Pirotte

**Affiliations:** 1 Centre Interfacultaire de Recherche en Pharmacochimie des substances naturelles et synthétiques, Laboratoire de Chimie Pharmaceutique, Université de Liège, 1 avenue de l'hôpital-CHU, B- 4000 Liège, Belgique; 2 Laboratoire d'Enzymologie Moléculaire et Fonctionnelle, Institut J. Monod, CNRS-Universités Paris VI et Paris VII, 2, place Jussieu, F-75251 Paris Cedex 05, France; 3 Département de Pharmacie, Université de Namur, FUNDP, 61 rue de Bruxelles, B-5000 Namur, Belgique; 4 Laboratoire de Biologie des Tumeurs et du Développement, Université de Liège, 1 avenue de l'hôpital-CHU, B-4000 Liège, Belgique

**Keywords:** coumarin derivatives, cell invasion, tumour growth

## Abstract

In search for new anticancer agents, we have evaluated the antiinvasive and antimigrative properties of recently developed synthetic coumarin derivatives among which two compounds revealed important activity: 3-chlorophenyl 6-acetoxymethyl-2-oxo-2*H*-1-benzopyran-3-carboxylate and 3-bromophenyl 6-acetoxymethyl-2-oxo-2*H*-1-benzopyran-3-carboxylate. Both drugs were able to inhibit cell invasion markedly in a Boyden chamber assay, the bromo derivative being more potent than the reference matrix metalloprotease (MMP) inhibitor GI 129471. *In vivo*, tumour growth was reduced when nude mice grafted with HT1080 or MDA-MB231 cells were treated i.p. 3 days week^−1^ with the bromo coumarin derivative. These effects were not associated with the inhibition of urokinase, plasmin, MMP-2 or MMP-9. The mechanism of action of the drugs remains to be elucidated. However, these two coumarin derivatives may serve as new lead compounds of an original class of antitumour agents.

The process of cancer invasion is a multistep event involving angiogenesis, local invasion, cell migration, dissemination and growth at a secondary site (Zetter, 1998). During malignant transformation, tumour cells have to acquire new functions such as self-sufficiency in growth signals, insensitivity to growth-inhibitory (antigrowth) signals, resistance to apoptosis, adhesive and invasive capacities (Hanahan and Weinberg, 2000). Basement membrane provides a main physical barrier to cell migration at different steps of this metastatic process: escape from the primary tumour in carcinomas, intravasation and extravasation during haematogenous dissemination. Invasion of basement membranes involves: (i) the adhesion of tumour cells *via* cell surface molecules, (ii) secretion of proteolytic enzymes including at least serine proteases and matrix metalloproteases (MMPs) and (iii) cell migration into target tissue in response to specific chemotactic stimuli (Liotta, 1986; Stetler-Stevenson *et al*, 1993; Hanahan and Weinberg, 2000). In addition, the acquisition of invasive properties by some tumour cells is associated with the alteration of several classes of proteins involved in cell–cell and cell–matrix interactions. These affected proteins include at least members of the immunoglobulin and calcium-dependent cadherin families and integrins (Hanahan and Weinberg, 2000). The proteolytic event induced by proteases allows the invasion of cancer cells locally and at distance, as well as the release or the activation of growth or angiogenic factors (for review, see Frankenne *et al*, 1999; Noël *et al*, 1999; Schmitt *et al*, 2000; Egeblad and Werb, 2002).

By using the Boyden chamber assay, we have evaluated different coumarin derivatives as anti-invasive compounds and analysed their inhibitory activity against some serine proteases and MMPs. In previous works, we have investigated a series of original coumarin derivatives (6-substituted 2-oxo-2*H*-1-benzopyran-3-carboxylic acid analogues). Some of them were potent inhibitors of serine proteases such as *α*-chymotrypsin (*α*-CT), human leucocyte elastase (HLE) and thrombin (THR) (Pochet *et al*, 1996,^2000^; Doucet *et al*, 1999). Based on the invasive assay, among eight coumarin derivatives evaluated, we have selected two compounds, 3-chlorophenyl 6-acetoxymethyl-2-oxo-2*H*-1-benzopyran-3-carboxylate (compound **4)** and 3-bromophenyl 6-acetoxy-methyl-2-oxo-2*H*-1-benzopyran-3-carboxylate (compound **7)** (Figure 1

**Figure 1:**
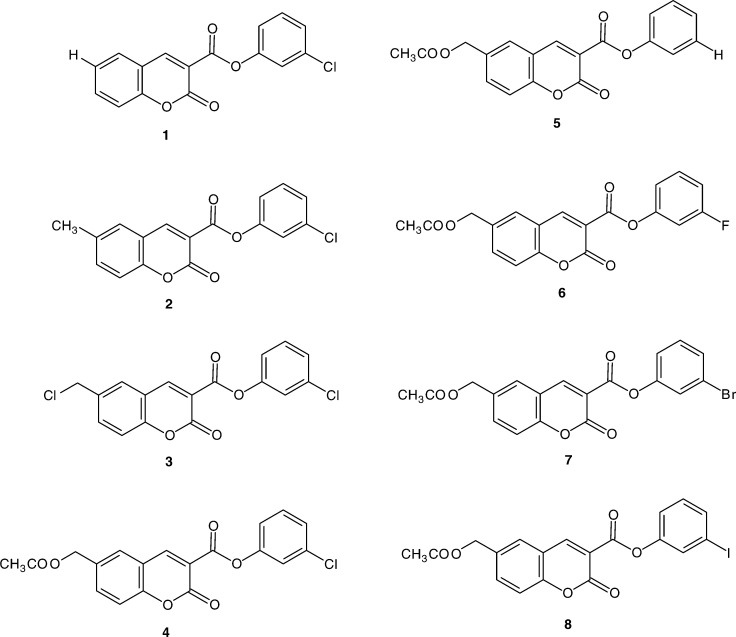
Chemical structure of the coumarin derivatives.

), which were further examined for their ability to affect the tumorigenicity *in vivo* of human fibrosarcoma and human breast adenocarcinoma cells. Their inhibitory potency against different serine proteases and MMPs was also determined. We provide evidence that these two coumarin derivatives display interesting and important *in vivo* antitumour effect.

## MATERIALS AND METHODS

### Chemistry

Melting points were determined on a Büchi-Tottoli capillary apparatus and are uncorrected. Infrared spectra were recorded as KBr pellets on a Perkin-Elmer 1750 FT spectrophotometer. The ^1^H nuclear magnetic resonance spectra were taken on a Bruker AW-80 (80 MHz) instrument in dimethyl-d_6_ sulphoxide with hexamethyl disilazane as an internal standard; chemical shifts are reported in *δ* values (p.p.m.) relative to internal HMDS. The abbreviations s (=singlet), d (=doublet) and m (=multiplet) are used throughout. Elemental analyses (C, H) were realised on a Carlo-Erba EA 1108-elemental analyser and were within ±0.4% of the theoretical values. All reactions were routinely checked by thin layer chromatography on silica gel Merck 60F 254.

### Synthetic coumarin derivatives

The coumarin derivatives were obtained according to previously described synthetic procedures (Pochet *et al*, 1996,^2000^). The physicochemical and spectral data of the new derivatives **6**, **7** and **8** are reported in Table 1

**Table 1:** Physicochemical data of the coumarin derivatives **6–8**

**Compound**	**Melting point (°C)**	**Yield (%)**	**Molecular formula**
**6**	170–172	53	C_19_H_13_FO_6_
**7**	141–142	55	C_19_H_13_BrO_6_
**8**	169–170	57	C_19_H_13_IO_6_

and Table 2

**Table 2:** Spectral data of the coumarin derivatives **6–8**

**Compound**	**IR (KBr) (cm^−1^)**	**^1^H NMR (80 MHz, DMSO-*d*_6_, HMDS) δ (p.p.m.)**
**6**	3066 (C–H aromatic); 1735 (C=O ester and lactone); 1629; 1580; 1486; 1363; 1247	2.05 (s, 3H, CH_3_), 5.1 (s, 2H, CH_2_), 7–7.95 (m, 7H, 5-H, 7-H, 8-H, 2′-H, 4′-H, 5′-H, 6′-H), 9.0 (s, 1H, 4-H)
**7**	3070 (C–H aromatic); 1735 (C=O ester and lactone); 1628; 1579; 1470; 1367; 1246	2.0 (s, 3H, CH_3_), 5.15 (s, 2H, CH_2_), 7.25–7.95 (m, 7H, 5-H, 7-H, 8-H, 2′-H, 4′-H, 5′-H, 6′-H), 9.0 (s, 1H, 4-H)
**8**	3065 (C–H aromatic); 1762 (C=O ester); 1722 (C=O lactone); 1623; 1575; 1466; 1376; 1248	2.1 (s, 3H, CH_3_), 5.1 (s, 2H, CH_2_), 7.15–7.9 (m, 7H, 5-H, 7-H, 8-H, 2′-H, 4′-H, 5′-H, 6′-H), 8.95 (s, 1H, 4-H)

.

### Cell culture

Human fibrosarcoma HT1080 cells and human breast MDA-MB 231 adenocarcinoma cells were maintained in Dulbecco's modified Eagle's medium (DMEM) supplemented with 10% (v v^−1^) fetal calf serum (FCS), penicillin–streptomycin (100 IU ml^−1^; 100 *μ*g ml^−1^), 2 mM glutamine and 10 mM HEPES buffer at 37°C in a humid atmosphere (5% CO_2_ and 95% O_2_).

### Coating of culture wells with type IV collagen

Plates (24-well) (Falcon, Becton-Dickinson, USA) and 6.5 mm polycarbonate filters (8 *μ*m pore size) of Transwell cell culture chamber inserts (Costar, Netherlands) were coated with 100 *μ*l of type IV collagen purified from human placenta diluted at 200 *μ*g ml^−1^ in phosphate-buffered saline (PBS). Coats were left to air-dry overnight in a laminar flow hood under UV light. Uniformity of the coating was assessed by Coomassie blue staining. Before use, coated plates and inserts were washed twice with water and incubated for at least 1 h in serum-free DMEM at 37°C (Maquoi *et al*, 1999).

### Invasion assay

HT1080 cells exponentially growing in T-75 flasks (Nunc, Costar, Netherlands) were collected by trypsin–ethylenediaminetetraacetic acid treatment, washed with serum-containing medium and allowed to recover from trypsinisation for at least 30 min at 37°C. Cells were then washed twice in serum-free DMEM and diluted in the same medium supplemented with 0.1% bovine serum albumin (BSA, fraction V, Sigma, USA) to a cell density of 6 × 10^5^ cells ml^−1^.

The influence of coumarin derivatives (0.1, 1 and 10 *μ*M) on HT1080 cell invasion and migration was assayed using Transwell cell culture inserts (Costar) and compared to the reference compound GI 129471 (1 *μ*M), an MMP inhibitor (Wheeler *et al*, 1998).

For chemoinvasion assays, type IV collagen-coated membranes (see above) were used, whereas uncoated membranes were employed for chemotaxis assays. Lower wells of chambers were filled with DMEM supplemented with 20% FCS and 1% BSA (fraction V, ICN) as chemoattractant, in the presence or absence of coumarin derivative (final concentration 0.1, 1 and 10 *μ*M) or GI 129471 (final concentration 1 *μ*M). Cells (6 × 10^4^) suspended in DMEM supplemented with 0.1% BSA (fraction V, ICN) were seeded in the upper wells. Chambers were subsequently incubated for 48 and 4 h for chemoinvasion and chemotaxis assays, respectively, in a humid atmosphere at 37°C (in order to maintain a chemotactic gradient, media from both lower and upper wells were renewed after 24 h). After incubation, filters were removed from the chambers, fixed in 4% paraformaldehyde for 15 min (4°C), permeabilised for 10 min in methanol (−20°C) and stained with Giemsa (4%) for 15 min. The upper surface of the filters was scrapped with a cotton swab and cells having reached the lower surface of the filters were visually counted using a light microscope (Vanox AH3, Olympus, Hamburg, Germany) at 20-fold magnification. Each assay was performed in triplicate. Results were expressed as percentages (±s.d.) of the migration of control cells.

### Cytotoxicity assay

In order to assess the potential influence of coumarin derivatives (0.1, 1 and 10 *μ*M) on cell metabolism, HT1080 cell suspension (6 × 10^5^ cells ml^−1^) was plated on type IV collagen-coated 96-well plates, 200 *μ*l well^−1^ (1.2 × 10^5^ cells), in the same culture conditions as described above and incubated for 48 h in the presence of different concentrations of the coumarin derivative (medium renewal occurred after 24 h). Media were subsequently harvested and replaced by 100 *μ*l of DMEM supplemented with 10% WST-1 (4-[3-(4-iodophenyl)-2-(4-nitrophenyl)-2*H*-5-tetrazolio]-1,3-benzene disulphonate, Boehringer Mannheim, Germany) and plates were incubated for 2 h at 37°C. Cellular metabolism was determined by measuring the absorbance of the medium between 420 and 480 nm (with a reference filter of 595 nm) on a microtitre plate reader (Multiscan MS, Labsystems, Finland). At the concentrations tested, the synthetic coumarin derivatives did not significantly affect cellular metabolism (data not shown).

### Proliferation and adhesion assays

Cells (6.8 × 10^4^ for proliferation assay and 1 × 10^5^ for adhesion assay) were plated on plastic or type IV collagen or matrigel in 24-well plates (Falcon, Becton Dickinson, USA). At each time points (2 h, 4 h, 7 h 30 min and 23 h for the adhesion assay; days 1, 2, 5 and 7 for proliferation assay), the medium was removed, and the cells were washed with PBS and frozen at −20°C. The DNA content was determined by spectrofluorimetry (Crescimano *et al*, 1996). For cell adhesion, the DNA content of the initial cell suspension was considered as 100%. Each assay was performed in triplicate.

### *In vivo* studies

Matrigel (basement membrane proteins) was extracted from the mouse Engelbreth–Holm–Swarm (EHS) tumour as previously described (Noël *et al*, 1991). Tumoral cells were detached by trypsinisation, harvested by centrifugation, resuspended in 0.25 ml of serum-free medium and mixed with an equal volume of cold matrigel (10 mg ml^−1^). The suspension (1 × 10^6^ cells injection site^−1^) was injected subcutaneously (s.c.) on both body flanks of 6–8-week-old female athymic nude mice (*n*=6; *nu/nu* Swiss mice from Iffa credo, L'Arbresle, France). The coumarin derivatives in PBS were injected intraperitoneally (30 mg kg^−1^) three times per week during the whole assay.

Animals received human care, and experiments have been carried out with ethical committee approval. The ethical guidelines that were followed meet the standards required by the UKCCCR guidelines (Workman *et al*, 1998).

The tumorigenicity defines the capacity of cells to induce tumour formation. The larger (a) and smaller (b) diameters of the apparent tumour were measured and served for tumour volume calculation according to *a* × *b*^2^ × 0.4 (Attia and Weiss, 1966). Injected mice were examined weekly. Tumours presenting a volume higher than 80 mm^3^ and which maintained a progressive growth were taken into account (Noël *et al*, 1992). Results are expressed as the mean (±s.e.m.) of the tumour volumes.

### Sodium dodecyl sulphate – polyacrylamide gel electrophoresis and zymography

The culture medium of human fibrosarcoma HT1080 cells (DMEM) was removed and concentrated 20-fold in a Vivaspin 6 ml concentrator. It was incubated for 30 min at ambient temperature in nonreducing sample buffer and run under Laemmli conditions (Laemmli, 1970) by loading a volume of 25 *μ*l onto 7.5% polyacrylamide gels containing 0.1% sodium dodecyl sulphate (SDS), 2 mg ml^−1^ casein and 0.16 *μ*M human plasminogen. Following electrophoresis, gels were incubated for 1 h in 2.5% (v v^−1^) Triton X-100 for removal of SDS and then for 1 h at 37°C in 50 mM Tris-HCl, 0.1 M NaCl, pH 7.6. Plasminogen activator (PA) activity was visualised as clear bands of lysis upon staining with 0.1% Coomassie blue R250. Purified human high-molecular-weight two-chain urokinase plasminogen activator (HMW-uPA) and low-molecular-weight urokinase plasminogen activator (LMW-uPA) were run in each gel as positive controls. An analogous protocol was followed to detect enzymatic activities towards MMP-2 and MMP-9 except that the 7% SDS–PAGE contained 1 mg ml^−1^ bovine gelatin. The gels were incubated at 37°C for about 18 h in 5 mM Tris, pH 7.6 containing 0.2 M NaCl, 5 mM CaCl_2_ and 0.02 (w v^−1^) Brij35. Protein staining was performed during 1 h with a 0.25% solution of Coomassie brillant blue R250 in 50% methanol and 7% acetic acid and destaining in a solution of 20% methanol and 50% acetic acid. Purified pro-MMP-2, pro-MMP-9 and MMP-2 were used as positive controls. MMP-2 was freshly prepared by incubating pro-MMP-2 with 100 *μ*M *p*-aminophenylmercuric acetate (APMA, dissolved in 50 mM Tris) for 30 min at 37°C. Some experiments were carried out with the culture medium after activation by 100 *μ*M APMA (30 min at 37°C). The effects of compounds **4** and **7** (1–100 *μ*M) incubated in the culture medium were checked for both types of zymography.

### Enzymatic studies

Bovine *α*-CT and LMW-uPA (MW=33 000 Da) were purchased from Sigma, HMW-uPA (MW=54 000 Da) from Choay, trypsin (TRY) and THR from Boehringer-Indelheim (Germany), and MMP-2 and MMP-9 from Calbiochem. Human leucocyte elastase and PM were from Elastase Products Co. and Biogenic, respectively. Except for THR, HMW-uPA, MMP-2 and MMP-9, enzyme concentrations were determined by active-site titrations using the appropriate titrant (Bender *et al*, 1966; Chase and Shaw, 1970; Powers and Gupton, 1977). Several enzymes were assayed spectrophotometrically at 405 nm using the following *p*-nitroanilide substrates: succininyl-Ala_2_-Pro-Phe-*p*-nitroanilide (Suc-AAPF-*p*-NA), benzoyl-Arg-*p*-nitroanilide (Bz-R-*p*-NA) and methoxysuccinyl-Ala_2_-Pro-Val-*p*-nitroanilide (MeO-Suc-AAPV-*p*-NA) for α-CT, TRY and HLE, respectively (obtained from Sigma); or S-2238 (H-D-Ile-Pro-Arg-*p*-nitroanilide), S-2444 (<Glu-Gly-Arg-*p*-nitroanilide) and S-2251 (H-D-Val-Leu-Lys-*p*-nitroanilide) for THR, HMW- and LMW-uPA, and PM, respectively (from Biogenic). Matrix metalloprotease-2 previously activated by treatment with APMA were assayed at 412 nm with the thiopeptide Ac-Pro-Leu-Gly-^S^leu-Leu-Gly-Oet in the presence of 5,5′-dithio*bis*(2-nitrobenzenic) acid (DTNB). The amidolytic activities of the enzymes were followed in 0.025 M sodium phosphate, 0.05 KCl, pH 7.5 for *α*-CT; 0.1 M HEPES, 0.5 M NaCl, 0.1% (v v^−1^) Tween 80, pH 8.0 for HLE; 0.01 M HEPES, 0.01 M Tris, 0.1 M NaCl, 0.1% (v v^−1^) PEG_6000_, pH 7.5 for THR; 0.1 M Tris, 0.01 M CaCl_2_, pH 7.5 for TRY; 0.025 M NaH_2_PO_4_, 0.1 M NaCl, 0.05% (v v^−1^) Tween 80, pH 7.5 for HMW-and LMW-uPA; 0.1 M NaH_2_PO_4_, 25% (v v^−1^) glycerol, pH 7.5 for PM; and 0.1 M Tris, 0.15 M NaCl, 0.01 M CaCl_2_, 0.005% (w w^−1^) NaN_3_, 0.03% (w w^−1^) Brij35, pH 7.5 for MMP-2 and MMP-9. All assays contained 10% (v v^−1^) DMSO and were run at 25°C in a Perkin-Elmer Lambda 5 or Kontron Uvikon 941 spectrophotometer equipped with a thermostated cell holder.

The kinetic parameters for the inhibition were determined either by the preincubation method for LMW-uPA and PM (small aliquots of the reaction mixture were withdrawn at intervals of time and the remaining activity determined), or by the progress curve method (HLE) when the inhibition was too fast to be measured accurately using the preincubation method. The minimum kinetic scheme for the inactivation process is described as follows:


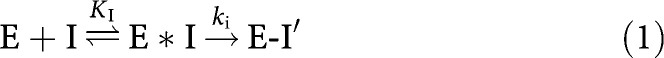


where E is the enzyme, E*I a kinetic chimere of the Michaelis complex and the acyl-enzyme, E-I′ the inactivated enzyme, *K*_I_ the apparent enzyme-inhibitor dissociation constant and *k*_i_ the inactivation rate constant at infinite inhibitor concentration. Using the preincubation method, the pseudo-first-order constants for inactivation *k*_obs_ were obtained from the least-squares analysis of semilogarithmic plots of remaining activity [E]/[E]_0_ against time (Equation (2)). They are related to *K*_I_ and *k*_i_ by Equation (3).


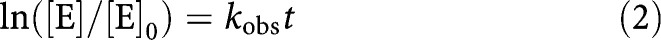



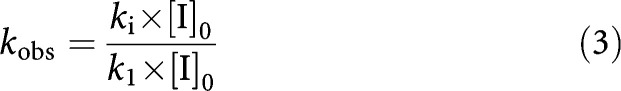


At low inhibitor concentrations, the ratio *k*_i_/*K*_I_ was obtained as *k*_obs_/[I]_0_. The enzyme, coumarin derivative and substrate concentrations were: [LMW-uPA]_0_=12 nM, [I]_0_=0.5–10 *μ*M, [S]_0_=30 *μ*M; [PM]_0_=28 nM, [I]_0_=2.8–28.3 *μ*M and [S]_0_=0.14 mM. In the progress curve method, the substrate competes with the inhibitor and the kinetic parameters are determined as previously described (Doucet *et al*, 1999). The enzyme, substrate and coumarin derivative concentrations were: [HLE]_0_=30 nM, [S]_0_=100 *μ*M and [I]_0_=0.05–70 *μ*M.

The products resulting from the spontaneous hydrolysis of compounds **1–4** and **7** obtained by 18 h incubation at pH 7.5 were also checked for a possible inhibitory effect against the purified enzymes MMP-2 and MMP-9 (50 pM and 1 nM, respectively; [S]_0_=220 *μ*M), LMW-uPA and HMW-uPA (12 and 15 nM, respectively; [S]_0_=30 *μ*M) and PM (17 nM, [S]_0_=270 *μ*M).

## RESULTS

### Synthetic coumarin derivatives reduce HT1080 fibrosarcoma cells invasion

**Figure 2:**
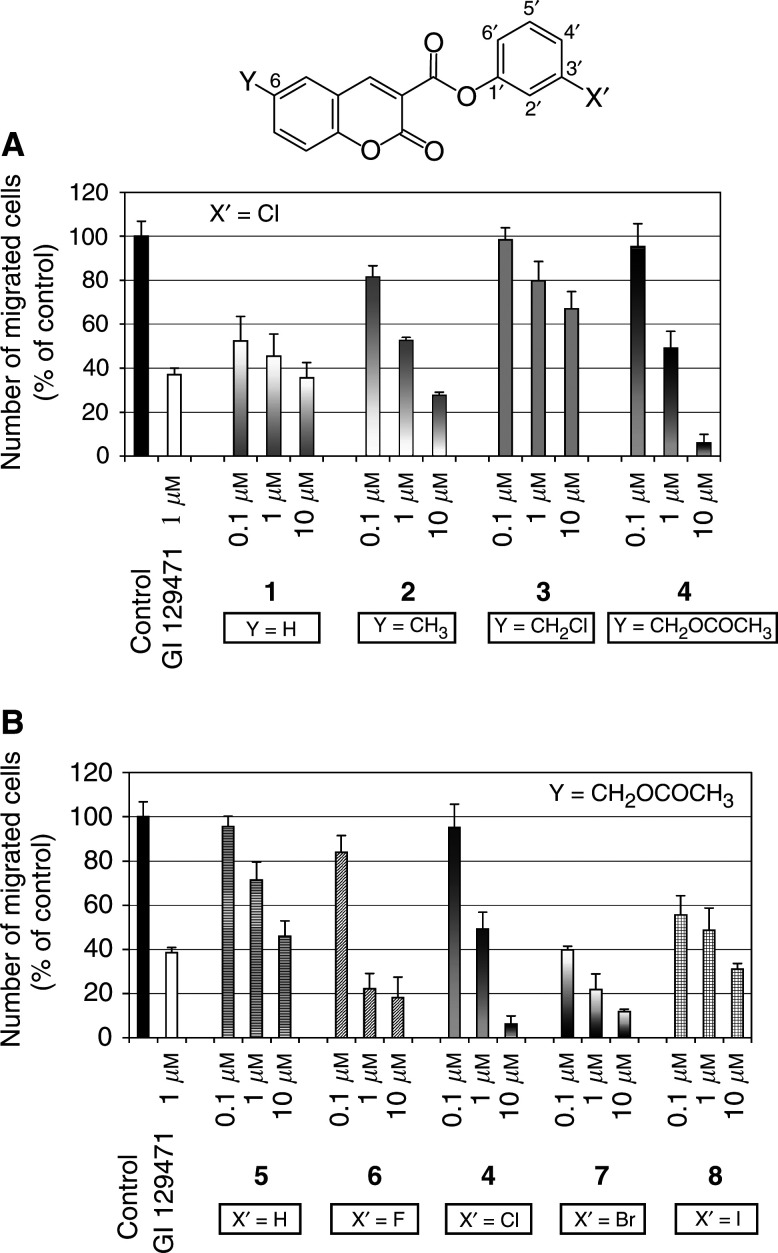
Synthetic coumarin derivatives reduce HT1080 fibrosarcoma cells invasion. HT1080 chemoinvasion was evaluated in Transwell cell culture inserts coated with type IV collagen. HT1080 cells (6 × 10^4^) were seeded in the absence (control) or presence of different concentrations of coumarinic inhibitor (0.1, 1 and 10 *μ*M) and the number of cells that have migrated was determined by visually counting the number of cells present on the lower side of the filters, as described in Materials and Methods: (**A**) 3-chlorophenyl 2-oxo-2*H*-1-benzopyran-3-carboxylate derivatives diversally substituted in the 6-position and (**B**) 6-acetoxymethyl-2-oxo-2*H*-1-benzopyran-3-carboxylic acid derivatives.

To determine whether coumarin derivatives could reduce the invasive behaviour of tumour cells, we measured the ability of cells treated or not with the coumarin derivative to pass through type IV collagen-coated Transwell cell culture inserts (chemoinvasion assay). Cell invasion in the absence of coumarin derivative was considered as 100% (Figure 2A and B

, Control). The results were compared to those observed with a well-known hydroxamate-MMP inhibitor: GI 129471, used at 1 *μ*M concentration as reference compound. In such a chemoinvasion assay, this reference compound led to a 60% inhibition of HT1080 cell invasion.

Coumarin derivatives **1–4** (Figure 2A) reduced HT1080 cell migration in a dose-dependent manner. The unsubstituted derivative **1** (Y=H) reduced the invasion by 50% at 1 *μ*M and 65% at 10 *μ*M. The 6-methyl **2** (Y=CH_3_) and the 6-acetoxymethyl **4** (Y=CH_2_OCOCH_3_) coumarin affected the invasion in a similar manner. At 1 *μ*M, both compounds led to a 50% inhibition, and at 10 *μ*M, a 70 and 90% inhibition were observed for the methyl **2** and the acetoxymethyl **4** derivatives, respectively. In contrast, cell invasiveness was not markedly affected by addition of the 6-chloromethyl-substituted derivative **3** (Y=CH_2_Cl) (only 30% invasion inhibition at 10 *μ*M). Thus, these data indicate that an acetoxymethyl group in the 6-position gave rise to better inhibition of cell invasion (Figure 2A).

We next synthesised the 3-phenyl (**5**) and the 3-halogenophenyl 6-acetoxymethyl-2-oxo-2*H*-1-benzopyran-3-carboxylate derivatives **6–8**. The different halogen atoms (F, Cl, Br or I) present on the 3′-position gave rise to different effects on cell invasion (Figure 2B). Addition of the unsubstituted derivative **5** (X′=H) has a lower inhibitory effect on cell chemoinvasion than that observed with GI 129471. Indeed, when this derivative was used at 1 *μ*M, a 30% inhibition of cell invasion was observed. At 10 *μ*M, it gave rise to a similar inhibition (50–60%) as that observed by using the reference compound at 1 *μ*M. Substitution by a halogen atom improved the inhibitory capacity of coumarin derivatives as compared to the nonsubstituted one. Addition of chloro (X′=Cl, compound **4**) and iodo (X′=I, compound **8**) derivatives at 1 *μ*M led to an inhibition quite similar to that observed with the reference compound. Interestingly, fluoro (X′=F, compound **6**) and bromo (X′=Br, compound **7**) derivatives appeared more potent, inducing an 80% inhibition at similar concentration. Moreover, chloro (compound **4)** and bromo (compound **7)** derivatives used at 10 *μ*M dramatically decreased the cell invasiveness (±90% invasion inhibition).

**Figure 3:**
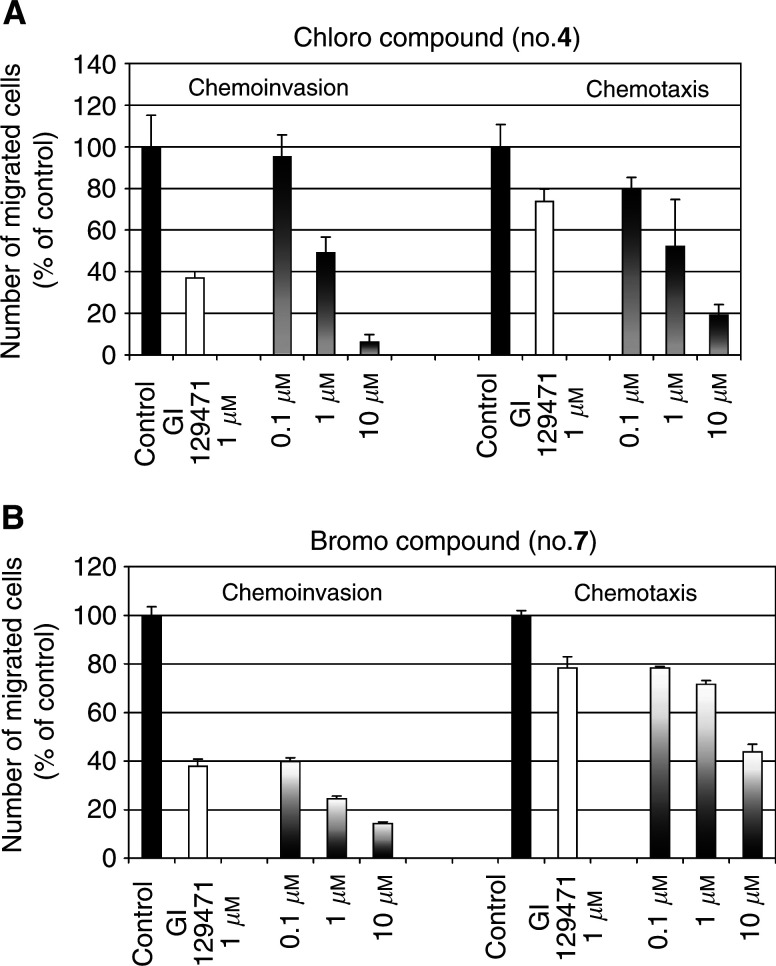
Effect of synthetic coumarin derivatives on HT1080 fibrosarcoma cell invasion. Chemoinvasion and chemotaxis were evaluated in Transwell cell culture inserts coated or not with type IV collagen, respectively. HT1080 cells (6 × 10^4^) were seeded in the absence (control) or presence of the coumarin inhibitor (0.1, 1 and 10 *μ*M) and the number of cells that have migrated was determined by visually counting the number of cells present on the lower side of the filters: (**A**) 3-chlorophenyl 6-acetoxymethyl-2-oxo-2*H*-1-benzopyran-3-carboxylate and (**B**) 3-bromophenyl 6-acetoxymethyl-2-oxo-2*H*-1-benzopyran-3-carboxylate.

Since these two products (compounds **4** and **7**) appeared to be very potent inhibitors of cell invasion, their anti-invasion and anticancer capacities were further studied *in vitro* and *in vivo*. We compared the ability of cells treated or not with the two selected coumarin derivatives to invade through type IV collagen-coated filters (chemoinvasion) and through uncoated filters (chemotaxis). Chemoinvasion corresponds to the capacity to invade through an extracellular matrix, while chemotaxis relies on the ability to migrate in response to a chemotactic factor. Addition of the chloro derivative **4** caused a potent and dose-dependent decrease of HT1080 cell chemoinvasion confirming the results reported in Figure 2B. In the chemotaxis assay, a similar dose response inhibitory effect was observed (20% inhibition at 0.1 *μ*M, 50% inhibition at 1 *μ*M and 80% at 10 *μ*M). In contrast, cell migration through uncoated filters was not markedly affected by the reference compound at 1 *μ*M (20–30% chemotaxis inhibition *vs* 60% inhibition of chemoinvasion) (Figure 3A

). Although the bromo derivative (compound **7)** reduced cell invasion and migration in a dose-dependent manner, this compound was a more potent inhibitor of chemoinvasion than chemotaxis (Figure 3B). Its effect on chemotaxis at 1 *μ*M was similar to that observed with the reference compound.

These effects of both compounds could not be ascribed to a modulation of cell adhesion or cell proliferation. Indeed, independent of the presence of coumarin derivatives, 100% of cell adhesion to plastic, type IV collagen or matrigel was reached within 7 h 30 min or 2 h, respectively. Similarly, cell proliferation was not affected by both compounds (data not shown).

### Enzymatic studies

**Figure 4:**
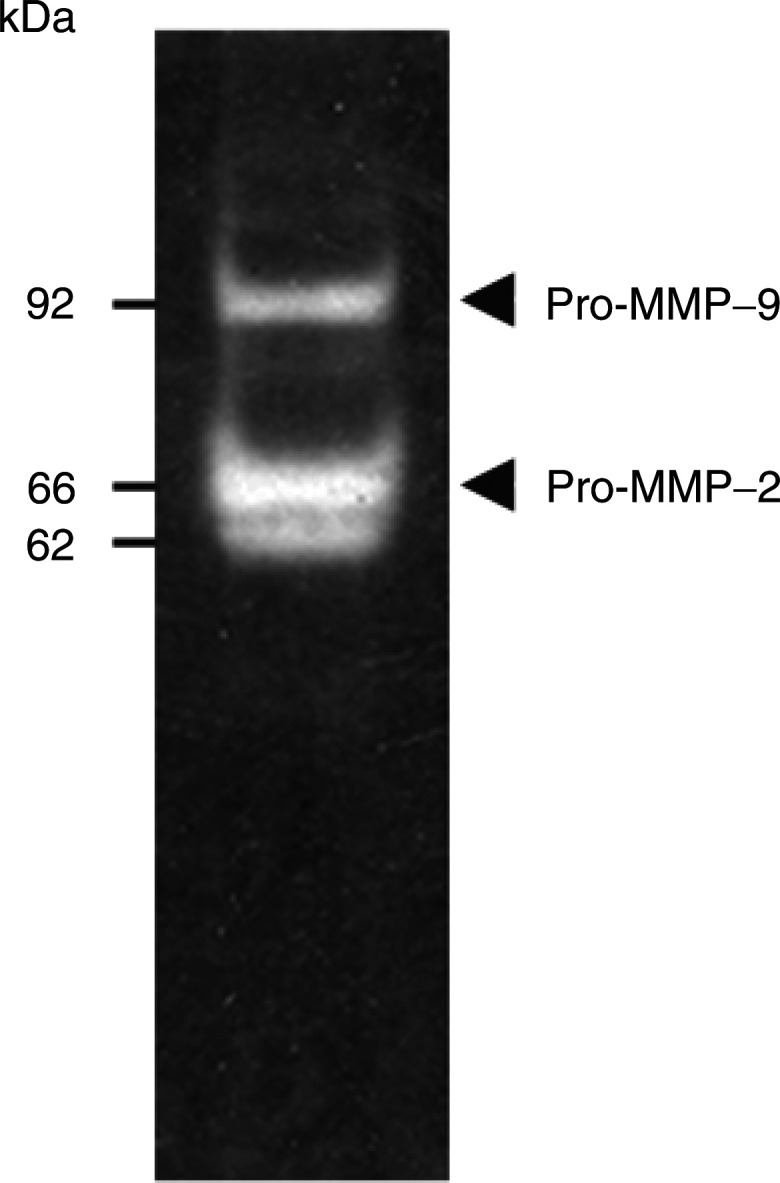
Matrix metalloprotease production by HT1080 cells analysed by gelatin zymography of the conditioned medium. The 59 and 120 kDa forms were present as traces.

To further gain insights into the characterisation of the selected coumarin derivatives tested (**4** and **7**), we have evaluated their activity against serine proteases and MMPs released by HT1080 cells. We first performed plasminogen and gelatin zymographic analysis on HT1080 cell conditioned media. Plasminogen activator activity was detected in all samples. Densitometric analysis of zymograms revealed greater amounts of HMW-uPA than LMW-uPA. Gelatin zymography demonstrated the presence of pro-MMP-2 (66 kDa), pro-MMP-9 (92 kDa), two MMP-2 activated forms (62 and 59 kDa) and a 120 kDa gelatinolytic species (Figure 4

). The latter activity was attributed to a complex formed between TIMP-1 and pro-MMP-9 (Maquoi *et al*, 1999). The addition of compounds **4** or **7** to the conditioned culture medium did not abrogate neither the HMW- and LMW-uPA nor the gelatinolytic activities.

Thereafter, we compared the inhibitory potencies of the two selected compounds to that of other coumarin derivatives against purified enzymes (Table 3

**Table 3:** Kinetic parameters for the inactivation of several serine proteases by 3-halogenophenyl 2-oxo-2*H*-1-benzopyran-3-carboxylate derivatives diversely substituted in the 6-position

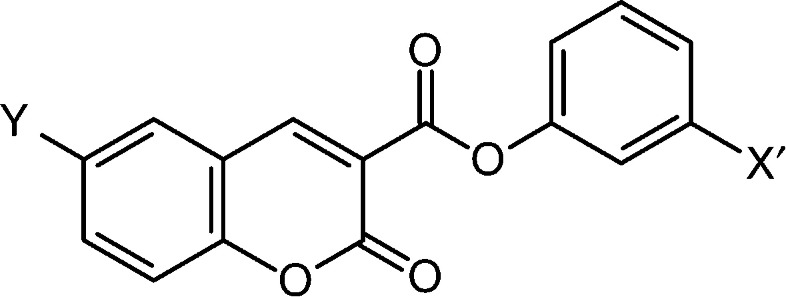						
			***k*_i_/*K*_I_ or k_obs_/[I] (M^−1^ s^−1^)**			
**No.**	**Y**	**X′**	**α-CT**	**HLE**	**LMW-uPA**	**Plasmin**
**1**	**H**	**3′-Cl**	<500^a^	610^a^	NI	ND
**2**	**CH_3_**	**3′-Cl**	9400^a^	1100^a^	NI	ND
**3**	**CH_2_Cl**	**3′-Cl**	762700^b^	630^b^	336	600
**4**	**CH_2_OCOCH_3_**	**3′-Cl**	20000^a^	1440^a^	NI	NI
**7**	**CH_2_OCOCH_3_**	**3′-Br**	ND	2190	NI	NI

NI=no inhibition, ND=not determined. High-molecular-weight two-chain urokinase plasminogen activator, MMP-2 and MMP-9 are not inhibited by compounds **4** and **7**. Standard errors are less than 15%. ^a^Data from Pochet *et al* (2000). ^b^Data from Pochet *et al* (1996).

). It was previously demonstrated that the 6-chloromethyl derivative (compound **3**) acted as a suicide substrate leading to enzyme irreversible inhibition of *α*-CT, whereas HLE was transiently inactivated through the formation of a stable acyl-enzyme (Pochet *et al*, 1996,^2000^). We found here that LMW-uPA and PM were inactivated by compound **3** (Table 3). No reactivation of inhibited LMW-uPA was observed either spontaneously after 18 h incubation or after treatment with NH_2_OH (pH 7.5, 25°C) demonstrating that the observed irreversible process is not because of the formation of a stable acyl-enzyme. Hydroxylamine is known to cleave acyl-enzymes (Doucet *et al*, 1999). It was verified that no noticeable denaturation of untreated LMW-uPA occurred during the above experiments. Presumably, compound **3** inactivates LMW-uPA according to the same mechanism as demonstrated for the inactivation of *α*-CT (suicide inhibition; Pochet *et al*, 1996). The closely related dihydrocoumarins (3,4-dihydro-3,4-dibromo-6-bromomethylcoumarin and 3,4-dihydro-3-benzyl-6-chloromethylcoumarin) were demonstrated to act as suicide substrates of LMW- and HMW-uPA with modification of the active site His57 (Reboud-Ravaux *et al*, 1982; Reboud-Ravaux and Desvages, 1984). In sharp contrast, purified LMW-uPA was not inactivated by compounds **1**, **2**, **4** and **7**. In addition, the activities of purified HMW-uPA, PM, MMP-2 and MMP-9 were not affected by the presence of compounds **4** and **7**, or of their hydrolysis products. Surprisingly, the 3-chlorophenyl 6-chloromethyl-2-oxo-2*H*-1-benzopyran-3-carboxylate **3**, which possesses an inhibitory potency against uPA and PM, did not markedly decrease the cell invasiveness. It is worth noting that the two compounds (**4** and **7**), which markedly decreased the cell invasiveness at 10 *μ*M (Y=CH_2_OCOCH_3_; X′=Cl and Br), were unable to act as uPA, PM or MMPs inhibitors.

### Effect of coumarin derivatives on tumour growth *in vivo*

**Figure 5:**
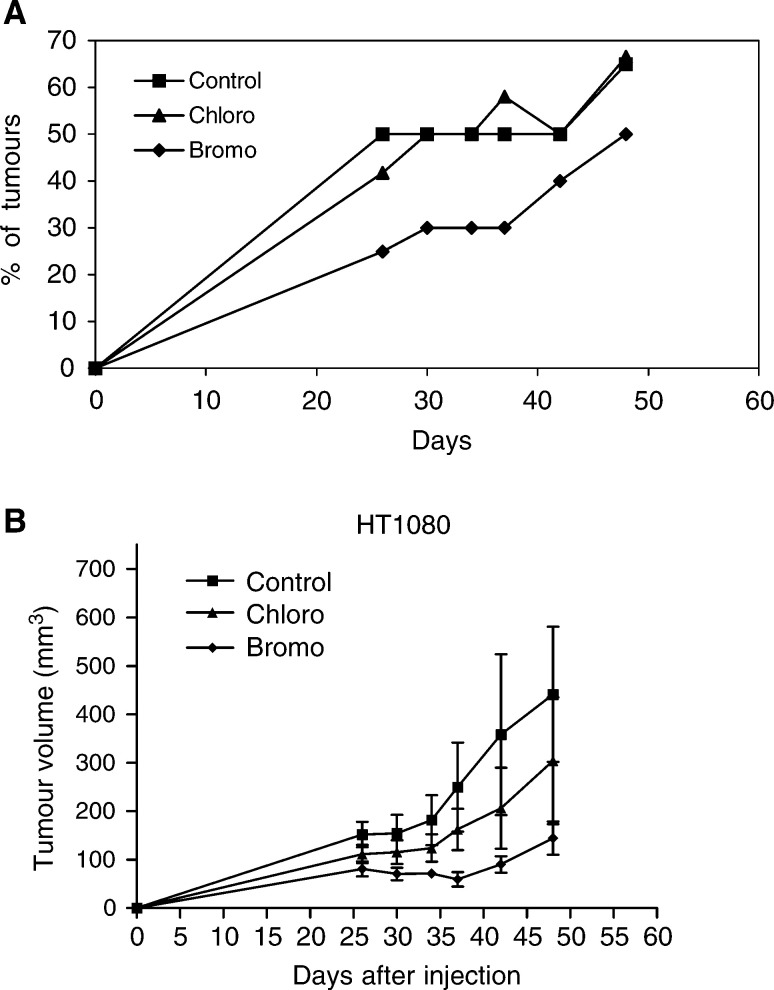
(**A**) Incidence of tumours induced by subcutaneous (s.c.) injection of HT1080 cells (percent of animals bearing tumour higher than 80 mm^3^). (**B**) Growth curves of tumour formed, in nude mice (*n*=6), after s.c. injection of HT1080 cells (1 × 10^6^) mixed with matrigel. The mice were examined twice a week for tumour appearance and measurement.

We next investigated the influence of the two selected compounds **4** and **7** (Y=CH_2_OCOCH_3_; X′=Cl and Br) on the development of tumours induced by the injection into nude mice of human breast adenocarcinoma and fibrosarcoma cells. Treatment of mice with bromo derivative (compound **7**) was found to reduce both the incidence (log Rank test, *P*<0.004) and growth of tumours induced by sc injection of HT1080 cells (Figure 5A and B

). After cell inoculation, a tumour incidence of 50% was reached after 26 days in control mice or animals treated with the chloro derivative, and after 48 days in animals treated with the bromo derivative (Figure 5A). These data demonstrate that the bromo derivative delayed the apparition of tumours. In addition, this compound significantly reduced the tumour growth (Figure 5B), since the tumour volume reached at the end of the assay was markedly reduced in animals treated with compound **7** (ANOVA analysis, *P*<0.05). In sharp contrast, the chloro derivative did not significantly affect tumour growth.

**Figure 6:**
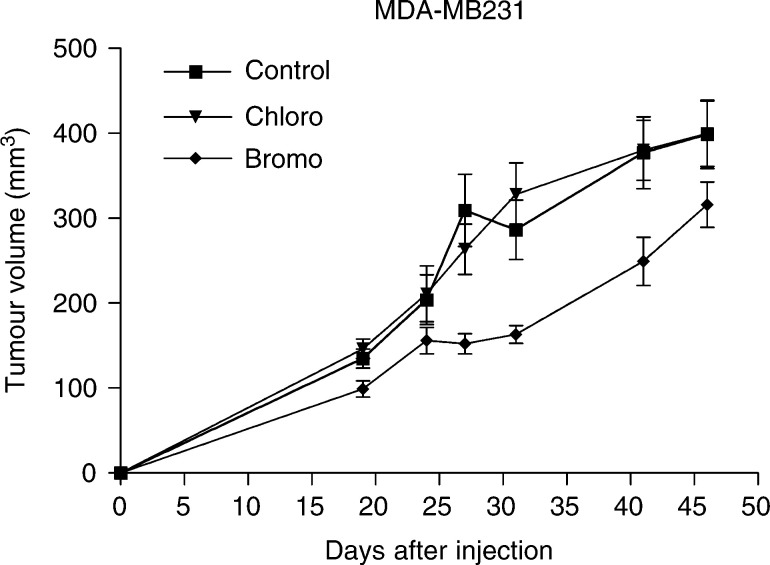
Growth curves of tumour formed, in nude mice (*n*=6), after s.c. injection of MDA-MB231 cells (1 × 10^6^) mixed with matrigel. The mice were examined twice a week for tumour appearance and measurement.

The effects of both compounds were also evaluated on the tumorigenicity of mammary MDA-MB231 cells. The MDA-MB231 cell inoculation led to 100% tumours within 2–3 weeks. While the bromo derivative did not affect tumour incidence, it again reduced significantly tumour growth (Figure 6

). (ANOVA analysis, *P*<0.05).

## DISCUSSION

This paper describes the evaluation of the anti-invasive properties of recently developed synthetic coumarin derivatives. The starting point of this work was the observation that 2-oxo-2*H*-1-benzopyran-3-carboxylic acid derivatives act as potent inhibitors of diverse serine proteases such as *α*-CT, HLE and THR (Pochet *et al*, 1996,^2000^; Doucet *et al*, 1999). Owing to possible implication of serine proteases such as uPA and PM in the metastatic process (Andreasen *et al*, 1997; Schmitt *et al*, 2000), a selection of previously described coumarins as well as new synthetic derivatives was therefore examined *in vitro* and *in vivo* on different experimental models. Thus, in order to highlight possible anticancer properties linked to their expected profile as protease inhibitors, the inhibitory capacity of the different compounds was determined *in vitro* on several serine proteases and MMPs (MMP-2 and MMP-9) (Egeblad and Werb, 2002). Moreover, chemoinvasion and chemotaxis *in vitro* assays as well as *in vivo* tumorigenic assays were conducted on nude mice, inoculated with HT1080 or MDA-MB231 cells.

Using noncytotoxic concentrations of compounds, our studies illustrated that some coumarin derivatives markedly inhibited the HT1080 cell invasiveness. The inhibition capacity varied according to the substituent present in the 6-position of the coumarin, and according to the nature of the halogen atom in the 3-position of the phenyl ring. In general, substitution by a halogen atom (particularly, a chlorine or a bromine atom) in the ‘meta’ position of the phenyl ring relative to the ester oxygen atom of 2-oxo-2*H*-1-benzopyran-3-carboxylate led to a better anti-invasive effect than that observed in the absence of any substituent. In the same way, an acetoxymethyl or a methyl group in the 6-position conferred an improved effect.

It is worth noting that 3-chlorophenyl 6-acetoxymethyl-2-oxo-2*H*-1-benzopyran-3-carboxylate (compound **4**) has almost the same potent inhibitory activity on cellular invasion (chemoinvasion) and on cell migration (chemotaxis). In sharp contrast, the corresponding 3-bromo derivative (compound **7**) led to a low inhibitory effect on chemotaxis, similar to that observed with GI 129471 used as reference compound (30% inhibition at 1 *μ*M). Interestingly, the bromo derivative was more potent in the chemoinvasion assay than the reference compound and the chloro-substituted analogue. These data emphasise the interest of the bromo derivative as an anti-invasive inhibitor. On the contrary, the chloro derivative which affected both chemoinvasion and chemotaxis is likely to modulate cell mobility in a manner independent of a protease inhibition. In accordance with these *in vitro* observations, our *in vivo* study in two experimental models demonstrates that only the bromo derivative markedly affected tumour growth. It reduced both the incidence and the growth of tumour induced by HT1080 cell injection. Furthermore, it affected the growth of more aggressive breast adenocarcinoma MDA-MB231 derived tumours.

The two selected coumarin derivatives **4** and **7** were not found to act as an uPA, PM, MMP-2 or MMP-9 inhibitor. Moreover, the coumarin derivative **3**, which was found to be a strong inactivator of *α*-CT and THR and which possessed an inhibitory potency towards uPA, did not markedly decrease cell invasiveness. Thus, the observed activity of the coumarin derivatives on HT1080 cell invasion could not be ascribed to their inhibitory activity against the enzymes tested. Both compounds were found to be unable to inhibit the angiogenesis evaluated in the aortic ring assay (data not shown) according to the procedure previously described (Blacher *et al*, 2001; Masson *et al*, 2002). Consequently, further experimental investigations are required to determine the exact mechanism of action of the bromo coumarin derivative and its biological target. For example, the recently described MT-SPS which are membrane-bound serine proteases implicated in tumorigenesis are possible targets for protease inhibitors like coumarins (Takeuchi *et al*, 2000; Hooper *et al*, 2001). In the light of our *in vivo* and *in vitro* results, the bromo coumarin derivative appears to be very promising as potential antitumoral agent. To the best of our knowledge, this study provides for the first time evidence that a coumarin derivative displays marked anti-invasive properties *in vitro* and antitumour activities *in vivo*.

The disappointment of MMP inhibitors in clinical trials as a result of the expression of a high toxicity (Coussens *et al*, 2002) emphasises the importance of a better elucidation of the mechanism of inhibition action *in vitro* and *in vivo*. Although coumarin derivatives might constitute an alternative to MMP inhibitors as anticancer agents, further biological investigations are required before any clinical trial.
